# A randomised controlled intervention trial evaluating the efficacy of a Mediterranean dietary pattern on cognitive function and psychological wellbeing in healthy older adults: the MedLey study

**DOI:** 10.1186/s12877-015-0054-8

**Published:** 2015-04-28

**Authors:** Alissa Knight, Janet Bryan, Carlene Wilson, Jonathan Hodgson, Karen Murphy

**Affiliations:** School of Psychology, Social Work and Social Policy, Alliance for Research in Exercise, Nutrition and Activity (ARENA), Bonython Jubilee Building, City East Campus, Frome Road, Adelaide, SA 5000 Australia; School of Medicine, The Flinders University of South Australia, Health Sciences Building, Sturt Rd, Bedford Park, Adelaide, SA 5042 Australia; School of Medicine and Pharmacology, The University of Western Australia, Stirling Highway, Crawley, Perth, WA 6009 Australia; School of Health Sciences, Alliance for Research in Exercise, Nutrition and Activity (ARENA), Bonython Jubilee Building, City East Campus, Frome Road, Adelaide, SA 5000 Australia

**Keywords:** Study protocol, Randomised controlled trial, Mediterranean diet, Age-related cognitive function

## Abstract

**Background:**

The incidence of age-related cognitive decline is rising considerably around the world. There is evidence from a number of recent cross-sectional and prospective studies indicating positive associations between the Mediterranean dietary pattern (MedDiet) and improved cognitive outcomes among the elderly including, reduced age-related cognitive decline and enhanced age-related cognitive performance. However, to date no study has validated these associations in healthy older adult populations (≥65 years and above) with randomised evidence. The main aim of the present study is to provide justified evidence regarding the efficacy of a MedDiet approach to safely reduce the onset of cognitive decline, and promote optimal cognitive performance among healthy older adults using rigorous, randomised intervention methodology.

**Methods/Design:**

MedLey is a 6-month, randomised controlled 2-cohort parallel group intervention trial, with initial assessment at baseline and repeated every three months. A sample of 166 healthy Australian men and women aged 65 years and above, with normal cognitive function and proficient in English language were recruited from metropolitan Adelaide, South Australia for the study. Participants randomly allocated to the experimental group are required to maintain an intervention dietary pattern based from the traditional Cretan MedDiet (i.e. vegetables, fruits, olive oil, legumes, fish, whole grain cereals, nuts and seeds and low consumption of processed foods, dairy products, red meat and vegetable oils) for six months, while those participants allocated to the control group are asked to maintain their customary lifestyle and diet. The primary outcome of interest is the quantitative difference in age-related cognitive performance, as measured by latent variables (cognitive constructs) sensitive to normal ageing and diet (i.e. speed of processing, memory, attention, executive functions, visual spatial and visuomotor ability). Secondary outcomes include change in biomarkers of inflammation, oxidative stress, lipid metabolism, glucose, insulin, blood flow velocity, and psychological well-being factors (i.e. stress, sleep, anxiety, depression).

**Discussion:**

To our knowledge this will be one of the first randomised clinical trials worldwide to provide evidence for the cause-effect relationship between the MedDiet and age-related cognitive function in a healthy older adult population (≥65 years and over).

**Trial registration:**

Australia and New Zealand Clinical Trials Register (ANZCTR): ACTRN12613000602729.

## Background

Age-related cognitive decline is rising as one of the world’s most detrimental health problems as the population ages. Although cognitive deterioration is considered an inevitable part of ageing, the rate and severity of cognitive decline is not homogenous among all individuals, ranging from normal age-related change, to mild cognitive impairment (MCI), to chronic neurodegenerative disease (e.g. dementia, Alzheimer’s disease [AD], Parkinson’s disease) [1]. Of utmost concern is the fact that pathological brain disorders such as AD are presently considered incurable, with no current lasting pharmaceutical treatment options that are effective in stopping or reversing the progression of disease. The efficacy of current treatments available (e.g. donepezil, galantamine, rivastigmine), at most, offer minor symptomatic relief for AD sufferers [2].

Recent evidence has provided emerging insight into modifiable lifestyle factors, such as diet, that may have an effect on slowing the onset and/or progression of age-related cognitive decline and promoting healthy cognitive ageing. In particular, the Mediterranean dietary pattern (MedDiet), rich in bioactive phytochemicals (e.g. constituents of olive oil, red wine, green leafy vegetables, fish, walnuts and seeds, such as antioxidants, fibre, vitamins, minerals, omega-3 fatty acids and monounsaturated fat), and low in red meat, dairy products and processed foods, is leading the interest of researchers and the general public. In fact, an accumulating number of empirical studies [3-5], including a recent systematic review [6] have shown preliminary evidence for the efficacy of MedDiet intake on a range of age-related cognitive outcomes. Collectively, such evidence indicates therapeutic potential for a MedDiet intervention to assist in protecting and preserving normal cognitive function in older age through slowing down the progression of cognitive decline and reducing risk of developing neurodegenerative pathologies [7].

The MedDiet approach to preserving better cognitive health in older age may be attractive to older adults, and also various governmental health agencies for a number of reasons. Firstly, the MedDiet is proposed as a potentially “natural” approach to reduce the onset of cognitive decline and prevent chronic neurological disorders (e.g. AD), as opposed to the alternative pharmaceutical approach. Improving cognitive function, life expectancy and overall health and well-being through diet and lifestyle modifications, may be a more favourable option for the elderly than using more stringent pharmacological alternatives. Indeed, many pharmacological treatments have associated side effects that can be quite adverse for this population [2]. Secondly, with reduced prevalence of these disorders, it is likely there will be a subsequent reduction in associated socio-economic and disability burdens as well.

### Cross-sectional and prospective studies

A growing body of cross-sectional and prospective studies have found higher adherence to a MedDiet pattern is associated with improvements in a range of cognitive outcomes for healthy older adult populations including: slower global cognitive decline [5,8,9], higher episodic memory [5], global cognitive performance [10-15], higher verbal memory performance [14], even after adjustments for cofounds. In addition, a number of studies [16-18] have shown evidence for these associations among non-clinical populations.

Although these findings appear encouraging, some studies report no beneficial effect of the MedDiet on age-related cognitive outcomes for healthy older individuals [19,20]. Thus, the question of whether adherence to a Mediterranean dietary pattern is beneficial to the normal (i.e. non-pathological) ageing brain remains equivocal at present.

### Randomised controlled trials

Without question, the only way of validating the efficacy of the MedDiet as a preventative approach for older adults will rest upon evidence from randomised controlled intervention trials (RCTs). However, to our knowledge, there has only been one published RCT on cognitive ageing outcomes among the elderly to date. Martínez-Lapiscina and colleagues [3] conducted a 6.5 year RCT in Spain, investigating the effects of a MedDiet intervention on global cognitive performance among 522 elderly participants at high risk of cardiovascular disease (i.e. had to be carrying the presence of type-2 diabetes, or at least 3 major risk factors hypertension, dyslipidemia, smoking and obesity). In a parallel-group, cardiovascular prevention trial, participants were randomised to either a MedDiet supplemented with extra virgin olive oil (EVOO), a MedDiet supplemented with mixed nuts, or a low-fat control diet, and assessed on global cognitive functioning using the Mini-Mental State Examination (MMSE) [21] and a Spanish version of the Clock Drawing Test (CDT) [22]. After 6.5 years of dietary intervention follow-up, results of this trial indicated that mean MMSE and CDT scores were significantly higher for participants allocated to a MedDiet pattern supplemented with either EVOO or mixed nuts, compared with the control group. This finding provides further support to the hypothesised benefit of MedDiet intake on age-related cognition.

However, the evidence generated by this study is restricted to a specific population (i.e. older adults at high cardiovascular risk), and the results may not be generalisable to the wider elderly adult population. The range of cognitive tests utilised in the study was restricted, preventing a clear delineation of those specific aspects of cognition assisted through the dietary intervention. Consequently, it is not possible to determine the nature and range of cognitive functions critically impacted (e.g. speed of processing, executive functioning, attention) and how these relate to changing biological and neurological functioning. This is an important gap to address in future RCTs because conclusions about the efficacy of a MedDiet pattern on change in age-related cognitive function cannot advance without generating a clearer understanding of how individual aspects of cognition, across all domains, are influenced by MedDiet intake during the normal ageing process [23].

### Underlying mechanisms of the Mediterranean dietary pattern on age-related cognition

To date, no clear mechanistic explanation has been documented regarding the inferred relationship between MedDiet intake and improved age-related cognitive function. However, preliminary evidence from prior observational studies has begun to shed some light on potential underlying factors that may be involved. Indeed, certain individual foods and their constituents found in the MedDiet pattern (i.e. vitamins E, B_6_, B_12,_ folate, monounsaturated fatty acids, fish, carotenoids, flavonoids, omega-3 and omega-6 polyunsaturated fatty acids and antioxidants) may be protective against the development of cognitive decline [24-26] possibly through a direct effect on brain physiology. When these foods and constituents are combined, it is assumed that the interrelated actions of a whole dietary pattern may generate even more powerful synergistic, additive or antagonist actions in the brain that single food factors are unable to achieve.

A second line of reasoning suggests biological mechanisms could also be implicated as underlying factors. Indeed, a growing body of studies have documented strong evidence showing the effect of nutrition on cardiovascular health, including the positive effect of the traditional MedDiet [27], and conversely, the inverse effect of the typical Western diet [28,29]. Of particular note, specific biological mechanisms such as oxidative stress, inflammation, insulin resistance and reduced cerebral blood flow have individually been postulated as key factors in the pathogenesis of both normal cognitive ageing and chronic neurodegenerative change [8,30-32].

Evidence that the MedDiet may reduce the risk of unsafe levels of these biological markers in the brain have been found in a number of recent studies. For example, Gu and colleagues [10] showed that adherence to a MedDiet pattern reduced inflammation markers C-reactive protein (*hs*CRP) and interleukin-6 levels. Similarly, the MedDiet has also been found to reduce oxidative damage [33], which may affect the physiology of the ageing brain directly, by protecting neuronal and cell-signalling function and maintenance. Other recent studies have shown benefits of MedDiet adherence on endothelial function. Sorond and colleagues [34] found that consumption of a flavonol-rich cocoa drink for two weeks significantly increased cerebral blood flow velocity among healthy older adults. Given that the MedDiet is particularly rich in flavonoids, there is a potential for similar effects to occur in the brain, improving blood flow and overall endothelial function [35].

Taken together, we propose the underlying mechanisms of the MedDiet pattern on age-related cognitive health may be multifactorial, with associations between MedDiet food components, cardiovascular risk factors, and age-related cognitive function. In turn, this complex event may generate either direct effects on brain physiology at the neuronal and cellular level, or influence cognitive function more indirectly through improved cardiovascular health.

## Methods

The Mediterranean diet for cognitive function and cardiovascular health in the elderly (MedLey) study is investigating a wide array of outcomes relating to age-related cognitive performance, cardiometabolic health, psychological wellbeing and genome health. The current paper provides a comprehensive synopsis for the main design of the MedLey trial, including ethics approval, participant recruitment, screening and selection, randomisation, study procedure, safety considerations, and focuses on the rationale and methodology for the cognitive outcomes of interest.

### Ethics

Ethical approval was given by the University of South Australia Human Research Ethics Committee, and the Flinders University Southern Adelaide Clinical Human Research Ethics Committee (ID:31163). The trial was successfully registered in the Australian New Zealand Clinical Trials Registry (ACTRN12613000636752), and adheres to the principles of the Australian Code for the Responsible Conduct of Research.

### Objectives for cognitive outcomes

The primary aim of the MedLey trial in relation to the cognitive outcomes was to comprehensively examine, over six months, the effects of a MedDiet pattern on age-related cognitive performance, specifically, speed of processing, memory, attention, executive functions, visual spatial and visuomotor ability. It was hypothesised that among a sample of non-demented, male and female Australian older adults aged 65 years and above, the MedDiet group would show significantly greater age-related cognitive performance in aspects of fluid cognitive ability when compared with the habitual dietary control group over six months.

Secondary aims were: 1. to explore whether the effect of MedDiet intake on age-related cognitive functioning is mediated by *hs*CRP (biomarker of inflammation), F_2_-isoprostanes (biomarker of oxidative stress in vivo), metabolic biomarkers (lipids, glucose, insulin), and blood flow velocity in the middle cerebral artery (MCA); 2. to determine whether psychological well-being factors (stress, sleep, anxiety, depression) moderate the strength of association between MedDiet intake and age-related cognitive performance.

### Study design

**Figure 1:**
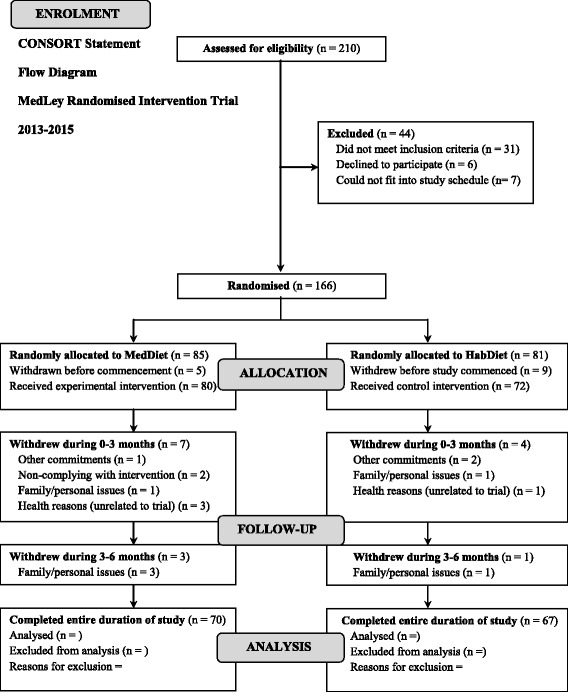
Flow diagram of the overall study design for the MedLey trial.

The MedLey study is a 6-month, randomised, controlled, 2-cohort parallel group comparison intervention trial, with repeated measures every three months, over three time points, to assess the effects of a MedDiet pattern with a habitual Western dietary pattern on age-related cognitive performance and cardiometabolic health. Due to the sheer extremity and length of the assessment process, a rolling recruitment was used over two cohorts (i.e. first cohort from June to September 2013, second from March to May 2014). Both cohort 1 and cohort 2 involved equivalent methods of recruitment, screening, assessment, and the same dietitians and researchers conducting the assessments and dietary consultations. Hence, collectively, the total study length of the trial involving the two cohorts was approximately 18 months in total. In accordance with the CONSORT statement [36], a flow diagram of participants intended progress throughout the intervention trial is documented (see Figure 1).

### Participant recruitment

Based on power and sample size calculations (see *Power* below), an initial sample of 166 healthy Australian men and women aged 65 years and above, with normal cognitive function and proficient in English language, were recruited from metropolitan Adelaide, South Australia through response to public advertisements in local broadcast and print (i.e. newspapers, magazines) media, posters, flyers and also presentations from our researchers at local senior groups and clubs. Participation in the MedLey trial was entirely voluntary, and participants were informed they were free to withdraw at any stage during the trial. Participants were provided with an honorarium of $100, to reimburse for study related expenses (e.g. parking fees).

### Participant screening and selection

Volunteers who expressed interested and responded to the study advertisements were sent an information pack and questionnaires to collect further information regarding their general health and medical history. The information pack included the following: a cover letter, participant information sheet including any risks associated with participation in the study, a pre-screening diet and lifestyle questionnaire (DLQ), map, and also a reply paid envelope. Upon receipt of volunteers returned, completed DLQ questionnaire, if they appeared to be eligible by the study investigators, they were invited to attend the Sansom Clinical Trials Facility at the University of South Australia for a screening visit.

The initial screening visit was used as a way to ensure that only volunteers who met the full inclusion criteria were included in the study. At this visit, volunteers signed a consent form and then subsequently had their weight, height and blood pressure measured, as well as providing a fasting blood sample for a full blood examination including: liver function, blood lipids, and glucose to ensure there were no underlying health concerns. Screening blood samples were analysed by SA Pathology, a National Association of Testing Authorities (NATA) accredited pathology.

As the population of interest for the MedLey RCT was healthy older adults (≥65 years), it was imperative to screen for MCI and dementia. A validated screening instrument; the DemTect [37] was chosen for this purpose. The DemTect includes five subtests: 1. a word list task to assess immediate and delayed recall, 2. a number transcending task, where participants translate numerals to number words and vice-versa (e.g. 100 to one hundred) to assess language, 3. a semantic verbal fluency task [items found in a supermarket task], and 4. a digit span task to assess working memory). Together, these subtests cover a diverse assessment of particular cognitive functions known to be sensitive to MCI and dementia, with high sensitivity in detecting early AD (sensitivity of 100%) and also in detecting MCI (sensitivity of 80%). Furthermore, the DemTect has been found to have high construct validity, inter-rater and test-retest reliability [37].

Scoring of the DemTect followed the standard protocol outlined by Kalbe and colleagues [37]. Firstly, each of the five subtest raw scores were adjusted for age and education. Subsequently, the five transformed subtest scores were summed together to provide an overall DemTect score for each participant, which was classified as either: ‘appropriate for their age’ (score 13–18), ‘mild cognitive impairment’ (score 9–12), or ‘suspected dementia’ (score ≤8). Those volunteers with scores <13 on the DemTect, indicating possible dementia, were kindly referred to a local general practitioner by one of the senior researchers.

Potential volunteers were invited to participate in the six month MedLey RCT if they met the following criteria: considered a free-living Australian man or woman aged 65 years and above, obtained a DemTect score ≥13 (no present dementia, healthy age-related cognitive function), and were proficient in English language. Participants were excluded from entry to the study if they scored an age adjusted score of <13 on the DemTect, had experienced any of the following conditions previously or currently which may cause some form of cognitive impairment, including a stroke, traumatic head/brain injury, or suffer a neurological or psychiatric condition, were using medications known to influence cognition, had current cardiovascular disease or angina, uncontrolled hypertension (>170/100), current or recent (in the last 6 months) malignancy, major liver, kidney, respiratory, gastrointestinal disease, a weight of ≥135 kg, were actively undertaking a weight loss program, using any form of appetite suppressant or Orlistat (Xenical), smoked, were already participating in another dietary intervention study, were vegetarian, and finally deemed by the investigator to be unwilling, unlikely or unable to comprehend or comply with the study protocol.

### Participant randomisation

Participants were de-identified according to gender, age and body mass index (BMI) and randomly allocated by a process of minimisation to one of two dietary groups Group 1. Mediterranean Diet experimental group (n = 85); Group 2. Habitual Diet, control group (n = 81). The two groups were then split into four groups comprising of two experimental and two control groups, with one experimental and one control group allocated to either cohort 1 or cohort 2 according to the preference and availability of the participants. Randomisation was performed by one of the chief investigators, who was not directly involved with study participants during data collection or data analysis. The researcher who administered the cognitive test battery and assessed and scored cognitive outcomes was blind to group assignment and will remain blind until after data analysis to reduce bias.

### Study procedure

Following the results of the initial screening visit, those volunteers who were eligible to take part were notified of their eligibility and invited to participate in the study.

Upon a signed declaration confirming consent, by the end of the trial, participants will have been assessed at three time points at the Alliance for Research in Exercise, Nutrition and Activity (ARENA) at the University of South Australia for the evaluation of age-related cognitive function and cardiometabolic outcomes (see Table 1).

**Table 1:** **Measurements for all outcomes of interest (cognitive and cardiovascular) investigated in the MedLey trial, undertaken at each study time-point**

**Assessment**	**0 months**	**3 months**	**6 months**	**Whole sample assessed**
Blood sample	✓	✓	✓	✓
Height	✓			✓
Weight	✓	✓	✓	✓
Cognitive function/psychological wellbeing	✓	✓	✓	✓
Waist circumference	✓	✓	✓	✓
Blood pressure and heart rate	✓	✓	✓	✓
Body composition and bone mineral density (DEXA)	✓	✓	✓	✓
Blood lipids	✓	✓	✓	✓
*hs*-CRP	✓	✓	✓	✓
Insulin & glucose	✓	✓	✓	✓
Urinary metabolites	✓	✓	✓	✓
CRTs	✓	✓	✓	✓
PGF2α	✓	✓	✓	✓
RBCFA	✓	✓	✓	✓
FMD	✓	✓	✓	✓
TCD	✓	✓	✓	✓
FFQ	✓	✓	✓	✓
3-day WFR		✓(2months)	✓(4 months)	
FCI		✓	✓	Half from cohort 1, all from cohort 2
Telomere length (qPCR)	✓		✓	20 males, 20 females
Mitochondrial DNA deletions (qPCR)	✓		✓	20 males, 20 females
DNA base oxidation (qPCR)	✓		✓	20 males, 20 females
APOε4 genotype	✓		✓	20 males, 20 females
Plasma oxidised and reduced glutathione	✓		✓	20 males, 20 females
Micronucleus index (Cytokinesis-block	✓		✓	20 males, 20 females
micronucleus cytome [CBMNcyt] assay)	✓		✓	20 males, 20 females

### Visit 1a, 2a, 3a (baseline, 3 months, 6 month)

Following an overnight fast, participants attend the ARENA on two morning visits (Visit a and Visit b), spaced one week apart, for assessments at baseline, three months and six months. On the first of the two week assessment visits (Visit a) participants commence a series of cardiometabolic measurements including: a collected 40 mL blood sample for the determination of glucose, insulin, *hs*-CRP, triglycerides, HDL-C, total cholesterol, F2-isoprostanes (PGF2α), erythrocyte fatty acids and plasma carotenoids, 5 mL saliva and buccal cells collected from both cheeks using a tooth brush for determination of DNA biomarkers, measurements of blood pressure and assessment of cerebral vasodilator responses (TCD) and endothelial vasodilatory function (FMD) (see Table 1).

In addition, on Visit (a), participants undergo neuropsychological assessment for the evaluation of age-related cognitive function. In this session, a battery of 13 cognitive tests are administered to participants individually, which takes approximately 1.5 hours to complete (see *Cognitive Measures* below). Tests are presented to each participant in the same order on each of their testing visits, along with standardised instructions. To reduce the possibility of practice effects, alternate forms of tests are used and counterbalanced across participants. Scoring of cognitive tests has been performed by the same researcher for all participants to reduce experimenter bias.

At the very end of Visit (a), participants are issued with a brown carry bag containing the following items to take home and complete during the following week: 1. blood pressure monitor to measure blood pressure at set intervals each day over the following week; 2. of a detailed, three day weighed food record (WFR), a food frequency questionnaire (FFQ) are used for the evaluation of habitual dietary intake consumed over the past 12 months 3. a large bottle and cup to collect a 24 hour urine sample; 4. a psychological well-being pack containing a series of questionnaires to evaluate level of depression, trait and state anxiety, stress and sleep quality (see *Psychological Well-being Measures* below). Explicit instructions are given to participants both in person and in a take home booklet to ensure assessment items are correctly administered and data is accurately recorded. Finally, at the completion of the session, participants are provided with complimentary breakfast.

Finally, from the mid-way point through baseline of cohort one and thereafter throughout the trial, the Food Cravings Inventory (FCI) (altered and approved protocol amendment) is administered at baseline, 3 months and 6 months to assess participants experience with food cravings and whether or not they are changing throughout the duration of the RCT.

### Visit 1b, 2b, 3b (baseline, 3 months, 6 month)

One week after Visit 1a, 2a, 3a, volunteers return to the ARENA centre for a second morning appointment (Visit 1b, 2b, 3b) after an overnight fast. Participants undergo assessments in height, weight, BMI, waist and hip circumference, and then complete a Dual X-ray Absorptiometry (DEXA) assessment, performed using a Lunar Prodigy DEXA machine by qualified investigators, to measure body composition, specifically precent of body fat. Participants are made fully aware of any potential risks associated with radiation exposure, and then asked to sign a consent form if they understand such risk, and are happy to proceed. Following obtained consent, participants are instructed to remove their outer clothing, jewellery, watches, and any other metal objects before lying in the supine position on the DEXA bed for the duration of the scan. After the DEXA scan is completed, participants are provide with a complimentary breakfast.

Following breakfast, participants meet with a qualified dietitian. On the first Visit (b) assessment appointment (Visit 1b), participants are informed which experimental dietary condition they were assigned to (i.e. MedDiet; experimental or HabDiet; control), and thereafter receive detailed instructions regarding their meal plan for the next 6 months. The meal plan for those participants randomised to the MedDiet is tailored specifically for each participant based on their individual tastes, preferences and energy requirements (see *Experimental Diet* below). To assist with making Mediterranean food choices which meet the specific guidelines of the MedDiet, participants are also issued with recipe books, menu guides and sample menus. Alternatively, those participants randomised to the HabDiet control group were asked to maintain their usual dietary and exercise pattern for 6 months.

Fortnightly visits with the dietitians are administer for all participants regardless of allocated diet group, to provide the opportunity to discuss any issues, questions, or receive further advice regarding their allocated diet. At week nineteen fortnightly visit for participants in cohort one, and week 5 for those in cohort two, a Lifetime Diet Questionnaire (LDQ) is administered to gather information about participants diet across the lifespan and whether there were differing or similar foods consumed during the different periods of their life.

### Safety considerations

The safety of participants is foremost, and ensured via procedures to minimise the risk of any harm in the form of physical pain or discomfort, emotional or psychological distress from either blood taking techniques, cardiometabolic measures, or cognitive testing. Should an adverse event arise due to participation in the trial it is treated immediately by trained ARENA staff where possible, with appropriate referrals provided and arranged for participants if needed. Blood samples are taken by ARENA staff and students trained and proficient in phlebotomy. This is done according to the ARENA standard operating procedure using aseptic techniques to minimise risk of infection to volunteers and injury to staff. All adverse events are reported in Case Report Form files, reported to the University of South Australia Human Research Ethics Committee at the time of the event, and followed up by the researchers.

## Measures

### Demographic information

Demographic information including: participant’s gender, age, self-rated physical, mental and social health status, marital status, highest level of education achieved, country of birth, parents’ country of birth, current occupation and level of work, income level, and perceived adequacy with current income level, family history of diagnosed chronic health conditions and diseases, including dementia, current medications, and vitamin supplements were gathered from participants for the ability to control for confounding variables.

### Dietary measures

#### Experimental diet - Mediterranean dietary pattern (MedDiet)

The Mediterranean style dietary pattern that was developed and used for the MedLey RCT, was based from the traditional Cretan MedDiet developed by Trichopoulou and colleagues [38], as well as the MedDiet food pyramids developed by Kromhout et al. [39] and Willett and colleagues [40]. In light of these literature sources, the key food and nutrient characteristics of the traditional Cretan Mediterranean dietary pattern (i.e. vegetables, fruits, olive oil, legumes, fish, whole grain cereals, nuts and seeds; moderate red wine, and low consumption of processed foods, dairy products, red meat and vegetable oils) were adopted as the primary foundation of the MedDiet. The approximate nutrient targets for per cent fat as monounsaturated fat, monounsaturated to saturated fat ratio, fibre, total contributions to energy from fat, saturated fat and carbohydrate and vitamin C, folate, and magnesium have been obtained from these sources, and are roughly in accordance with previous dietary intervention trials investigating the MedDiet [41,42].

Throughout the duration of the intervention, participants who were allocated to the MedDiet group received sufficient food, provided in appropriate cooler bags for transport, which incorporated 30-35% of the total energy provided by their allocated diet. Such foods included: Legumes (chickpeas, lentils, four-bean mix) (kindly donated by Simplot Australia Pty. Ltd), yoghurt (natural/flavoured Greek), Australian extra virgin olive oil (kindly donated by Cobram Estate), canned tuna (kindly donated by Simplot Australia Pty. Ltd.), walnuts, almonds (kindly donated by The Almond Board of Australia) and peanuts (kindly donated by the Peanut Company of Australia).

#### Control diet - Australian habitual dietary pattern (HabDiet)

Participants randomly allocated to the control group (HabDiet) are asked to maintain their usual lifestyle and dietary pattern. To reduce bias, on a fortnightly basis, they are also required to attend the ARENA clinic to meet with the dietitian to have their weight measured, discuss any changes in medication and any issues that may have arose. Volunteers are provided with either a food voucher for their supermarket of choice, or sufficient food provided throughout the intervention trial, that account for around 20-30% of their estimated energy requirements in the form of dietary staples, including: canola oil (kindly donated by Goodman Fielder Ltd), bread, milk and yogurt if it is part of their habitual diet.

#### Compliance

Compliance is measured via four means of assessment, 1. a semi-quantitative daily food check list, 2. the food frequency questionnaire (FFQ; Cancer Council Victoria), 3. the 3-day WRF's, 4. red blood cell fatty acid RBCFA composition (i.e. marker of fat intake which will be important given changes in saturated fat/monounsaturated fat intake), 5. plasma carotenoids (CRTs) via a blood sample, as biomarkers of fruit and vegetable intake, and 6, urinary metabolites. Participants are assessed on all means of compliance at each assessment time-point to ensure a high level of compliance is met. Furthermore, participants are continually instructed and monitored by the fully qualified dietitians on the trial each fortnight to make certain compliance is followed accordingly.

### Cognitive measures

The neuropsychological test battery for the MedLey RCT includes a series of individual cognitive measures to assess a diverse range of particular cognitive functions sensitive to normal age-related effects, and also likely to be affected by dietary factors. All included tests are administered via a paper- and -pencil format, in a private room, with only one participant and the examiner at any given time. The rationale for the selected test battery was based on sensitivity to ageing with healthy older adult populations (65–90 years), and hypothesized effects on the brain. Furthermore, in an attempt to achieve the highest standard of psychometric quality possible, the range of cognitive measures chosen for the MedLey RCT were selected using a highly authoritative taxonomy of cognitive measures by Lezak [43].

### Executive function

#### Interference control

Dodrill’s [44] version of the Stroop Test is used to assess participant’s level of interference control (i.e. cognitive form of inhibition and selective attention). This version of the Stroop Test consists of two alternate trials using one stimulus card that contains a list of 176 colour words printed in incongruous ink (e.g. the word “blue” printed in yellow ink), and randomly ordered. In the first trial participants are asked to read the words while ignoring the colours they are printed in, as quickly as they can, going across the page line by line. In the second trial, participants are asked to name the colours in which the words are printed while ignoring the words themselves. Scores are based on the time taken to complete each task, and also the time taken to reach the half way point. As standard, two performance scores are generated for the Stroop Test; the time taken to complete each trial. From these scores, an interference score is then calculated, which represents the ratio of the time taken to name colours divided by the time taken to read words. Higher scores reflect higher levels of interference and therefore poorer performance. The additional time taken to perform the second subtask compared with the first subtask is commonly referred to as the “Stroop interference effect”, and reflects an individual’s degree of interference control. In addition, the number of corrected and uncorrected errors produced by the participant is also collected for the ability to assess the severity of impaired performance on this test [45]. The Stroop interference score has been found to correlate moderately well with other measures of attention involving continuous performance tasks (*R*^2^ = .310 [46], providing evidence for construct validity. Furthermore, good test-retest reliability (*r* = .90) has been documented for this version [47]. Alternate forms of this test was used and counterbalanced across participants over the three treatment arms to reduce potential practice effects.

#### Strategic retrieval search

Two measures of verbal fluency, namely the Initial Letter Fluency (ILF) and the Excluded Letter Fluency (ELF) tests, are administered. In the ILF [43] participants are asked to produce as many words beginning with a particular letter as quickly as they can in 60 seconds, without using words that begin with a capital letter for a brand, place or person (e.g. for the letter *C* “Canberra or Christine”), words that are numbers (e.g. “eight”), or the same word that ends with a different ending (e.g. “catch, catching”). Finally, participants must name words that contain more than three letters. Participants are asked to repeat this process for two letters (for *F* and then for *S*) or (*L* and *C,* alternate form), on two separate 60 second trials. A total ILF performance score on this task represents the number of words correctly produced across the two trials, summed together. Bryan and colleagues [48] found a moderate alternate form reliability coefficient (*r* = .72) for the ILF test. In the ELF, participants were asked to produce as many words as possible that do not contain the letter *E* (on the first trial, or *R*, alternate form), and then not containing the letter *A* (on the second trial, or *T*, alternate form). Scores on both trials were then summed together to produce an overall ELF score. Evidence for moderate alternate form reliability for the ELF (*r* = .61) was found by Bryan et al. [48]. Alternate forms of both the ILF and the ELF were used and counterbalanced across participants over the three treatment arms.

#### Planning

D-KEFS [49] version of the Tower of London (TOL) test is used to measure executive functions, particularly spatial planning (i.e. the ability to problem solve in a way that involves mentally visualizing the outcome prior to physically carrying out the action) [49], as well as inhibition and rule learning. In a series of nine different problems, participants are asked to move a set of five disks from a starting configuration, which has three vertical pegs on top of a wooden platform. The aim is to move the set of disks to the specified goal configuration in the least number of moves possible, while overcoming two specific restrictions (i.e. a larger disk may not be placed on top of a smaller disk and only one disk can be moved at a time). Scores on the TOL represent the total number of permitted moves made across all the items relative to the minimum number of moves required to solve the problems, the number of violations (i.e. illegal moves) made, and also the total time taken to complete the tasks, which provides an index of an individual’s spatial planning ability, and learning strategy ability. Taken together, fewer moves used to successfully construct the towers of items 1 through to 9, and less time taken, represents greater executive functioning ability. High internal consistency for The D-KEFS Tower Test among older adults has been found by Delis, Kaplan and Kramer [49], with intercorrelations between test items ranging from .61 to .72.

### Memory

#### Episodic recall and recognition

Rey and Schmidt’s [50] Rey Auditory Verbal Learning Test (RAVLT) is used to assess episodic verbal recall and recognition. In the first trial, the examiner reads a list of 15 words (List A) at a rate of one word per second and asks the participant to repeat back as many words as they can remember, in any order. The examiner then reads the same list of 15 words aloud and asks the participant to again recall the list of words. This process continues for trials 3, 4 and 5. In trial six, the examiner reads aloud a second list of 15 different words (List B), and asks the participant to repeat back as many words as they can remember. Immediately after recall of List B, participants are then asked to recall as many words from List A as they can remember. In trial seven, following a 20 minute delay, the examiner then asks the participant to recall as many words as they can remember from List A. Finally, the participant is shown an A4 sheet of printed words that contained a total of 30 words from both List A and B, as well as 20 distracter words not appearing in either list. They are then asked to circle the words they identified as being read aloud, and also indicate whether they were from the first or second list of words by placing a “1” or “2’ next to the words they have circled. RAVLT performance scores consisted of the sum of words recalled from the five trials of List A, words recalled after a delay, and those recognised. The validity of the RAVLT in identifying memory impairment has been shown by Tierney and colleagues [51] who found healthy elderly participants performed significantly better on all measures of the RAVLT compared with demented patients with either Parkinson’s disease or Alzheimer’s disease. Evidence for good test-retest reliability for the RAVLT has been found by Knight, McMahon, Green and Skeaff [52]. Alternate forms of RAVLT (A, B and C) was used and counterbalanced across participants over the three treatment arms.

#### Short-term memory and working memory

The Digit Span Forward (DSF), Digit Span Backward (DSB) and the Letter-Number sequencing (LNS) subtest tasks from the latest version of the Wechsler Adult Intelligence Scale (WAIS-IV) [53], are used to measure short term memory (DSF) and working memory (DSB and LNS). For DSF, participants are read a consecutive string of numbers (from 1–9) at a rate of one per second, and asked to repeat them back in the exact order they are presented. For the DSB task, participants are asked to repeat the sequence of numbers back in the exact reverse order that they were presented. String lengths range from two to eight digits and each trial presented two lists of the same string length. For each one of the eight trails over the two Digit Span tasks, a score of zero is given if a participant makes an incorrect response, and a score of 1 was given for a correct response. Individual total raw scores for the DSF and DSB tasks is calculated by summing the accumulated scores for the eight trials administered to each separate task. The maximum total raw score for both the DSF and the DSB was 16.

For the LNS task, participants are presented with strings of numbers and letters at a rate of one item per second, and afterwards asked to repeat back the numbers in numerical order followed by the letters in alphabetical order. String lengths ranged from two to eight digits and letters, and each trial presented three lists of the same string length. Following standard scoring procedures as outlined in the test manual [53], all seven trials (with three test items per trial) (not including sample items) were given a score of 0, 1, 2 or 3 (0 = fails all three trials, 1 = passes one trial, 2 = passes two trials, 3 = passes all three trials). For trials one and two, a score of 0 is given if a participant provides an incorrect response, suggesting they did not know the answer, or did not provide an answer within the allocated time limit of 30 seconds. For trials three to ten, credit is given for providing the correct letters in sequence followed by the correct letters in sequence.

Psychometrically, the DSF and the DSB tasks of the Digit Span subtest of the WAIS-IV [53] have been reported to have good to excellent internal consistency reliability (*r* = .81 for the DSF, and *r* = .93 for the DSB) for older adult populations [54]. A confirmatory factor analysis of the WAIS-IV (model 5) revealed the Digit Span subtest loaded onto the Working Memory factor .76, suggesting the internal structure of the test captures the content area reasonably well. Letter-number sequencing from the WAIS-IV has been reported to be a reliable subtest (*r* = .88), and shown correlate better with the Digit Span subtest than any other subtest of the WAIS-IV, indicating it is indeed measuring memory [55].

#### Visual-spatial memory

The Benton Visual Retention Test (BVRT) [56] has been employed to assess visual-spatial memory ability, visual-motor construction ability and visual perception. Following standard administrative and scoring procedure [56], participants are shown 10 consecutive geometric designs for 10 seconds each. Once the participant has had 10 seconds viewing time, the design is hidden from view and they are instructed to reproduce the design from memory by drawing it on paper. Performance is scored as described in the test manual [57] with two generated outcomes; 1. the total number of correctly reproduced designs (0–10), and 2. the total number of errors produced (0–10) that fall under six categories: distortions, rotations, omissions, preservations, size, and misplacements. Psychometrically, the BVRT has been found to have good discriminant validity in the way it distinguishes dementia from the effects of normal ageing [58]. Moderate test-retest reliability for the Expected Number Correct Score (*r* = .78), along with a relatively low test-retest reliability coefficient for the Error score (*r* = .57) has been documented by prior research [59]. Alternate forms of the BVRT (C, D and E) was used and counterbalanced across participants over the three treatment arms.

#### Speed of processing and visual-motor coordination

The Symbol Search and Coding core subtests from the WAIS IV [53] were employed to measure processing speed. The Symbol Search task involves participants scanning a search group of symbols and indicating whether one of the symbols in the target group matched. If a match was found, the participants are instructed to draw a line through the shape. Alternatively, if there was no match, the participant is instructed to draw a line through the NO box. There are 60 items in this test, and participants are asked to complete as many as possible within 120 seconds. Symbol Search subtest has 60 items and participants are asked to complete as many as possible within 120 seconds. The total raw score for Symbol Search is calculated as standard [53] by subtracting the total number of incorrect responses from the total number of correct responses (maximum score = 60).

The Coding subtest has 135 items and requires participants to work within a time limit of 120 seconds. Participants are asked to copy symbols that are paired with numbers in the correct order, as quickly as possible, without making mistakes or skipping any. Participants are given one point for each correctly drawn symbol made in the allocated time limit of 120 seconds, not including sample items. The total raw score for Coding is calculated as the number of symbols correctly drawn within the 120 second time limit (maximum score = 135). Numerous studies documented in the Technical and Interpretative Manual [53] examining the psychometric properties of the WAIS IV’s Symbol Search and Coding subtests, support the validity and reliability of these measures. The test items within the Coding and Symbol Search subtests correlate highly with each other, and also load onto the “Processing Speed” factor better than any other subtest in the WAIS-IV (i.e. Coding .83 and Symbol Search .77) suggesting they adequately reflect the construct. Test-retest reliabilities reported by Wechsler [53] for Symbol Search range from .75 to .84, and from .81 to .86 for Coding.

### Psychological well-being measures

Evidence from past research has indicated that depression and anxiety [60] stress [61] and sleep [62] can all individually affect cognitive function. Thus, it was deemed important to determine whether any differences between the two dietary groups could result from differences in these variables. Therefore, depression, anxiety, stress and sleep quality are assessed in the MedLey RCT to control for potential confounding variables.

The following psychological well-being instruments are utilised in the current study: 1. the Spielberger State-Trait Anxiety Inventory Form Y (STAI-Y) [63] to measure symptoms of anxiety; 2. the Perceived Stress scale (PSS) [64] to measure subjective stress levels; 3. The Centre for Epidemiological Studies Depression Scale (CES-D) [65] to measure symptoms of depression; 4. a modified version of the Leeds Sleep Evaluation Questionnaire (LSEQ) [66] to assess the quality of their previous night’s sleep. These instruments were selected on the basis of their sound psychometric properties and sensitivity with older adults. For example, the STAI-Y has been found by Barnes, Harp and Jung [67] to have excellent test-retest reliability (*r* = .88) at multiple time points; evidence for excellent internal consistency reliability (*r* = .91) has been found for the PSS [68]; good internal consistency reliability (*r* = .88) has been found for the CES-D [69]; and Parrott and Hindmarch [66] have demonstrated with factor analysis, the four-factor structure of the LSEQ were correlated.

### Cardiometabolic measures

#### Biomarkers for oxidative stress, inflammation, insulin resistance, glucose regulation

Fasted 40 mL blood samples are collected from participants at each treatment arm (i.e. visit 1b, visit 2b, visit 3b) to assess *hs*CRP (biomarker of inflammation), F_2_-isoprostanes (biomarker of oxidative stress), plasma glucose and insulin biomarker levels, for the ability to detect metabolic abnormalities that may reflect insulin resistance.

All blood samples are processed according to the Australian standard operating procedures (SOPs). Blood samples have been, and will continue to be taken by venepuncture, by qualified personnel who have been trained using standard aseptic techniques to avoid cross-infection. Thereafter, blood samples are transported on ice to SA Pathology on Frome road in Adelaide for analyses. F2-isoprostanes samples are stored at -80C until sent to the University of Western Australia (UWA) for the analysis of oxidative stress.

#### Cerebral blood flow velocity - transcranial doppler (TCD)

Transcranial Doppler (TCD) ultrasound methods are used to measure changes in middle cerebral artery blood flow velocity (MCAV) at rest and in response to changes in 5% CO_2_ inhalation (hypercapnia-induced cerebral vasodilation), to determine the effects of dietary intake of a MedDiet and HabDiet pattern on cerebral vascular responsiveness to CO_2_. The examination takes place in a temperature controlled room, where participants are sitting in a comfortable upright position on a chair throughout the entire assessment. A mouthpiece with a two-way, non-rebreathing valve is fitted for each participant and a nose clip blocks inhalation through the nose. Once secure, participants are asked to inhale a gas mixture of 5% CO_2_/95% O2 for 2.5 minutes to initiate hypercapnia induced cerebrovascular dilatation. The left transtemporal window (i.e. between the left ear and alongside the zygomatic bone) is used for insonation of the MCA. Assessments of the mean flow velocity in the MCA is then recorded at a depth of 50 ± 10 mm for each cardiac cycle throughout the entire 180 seconds, and performed three times with a two minute rest period, for optimum test reliability. A fitted spline curve is used to smooth data collected during hypercapnia, which provides the ability to determine the peak increase in mean flow velocity. MCAV is expressed as a percentage change from baseline [(peak mean flow velocity-baseline mean flow velocity) * 100/baseline mean flow velocity].

#### Data screening and statistical analysis

Analyses will be conducted using SPSS Version 21.0 for Windows (Chicago, IL, USA), with an alpha set at p < .05. Following an “intention to treat” basis, descriptive statistics will be performed for each testing phase with respect to baseline characteristics. For categorical variables, frequencies and percentages will be determined and provided, along with the mean, standard deviation (SD) and the standard error of the mean (SEM) statistics for the continuous variables (or non-parametric equivalents). Any non-normally distributed variables will be transformed in accordance with standard methodology to obtain a normal distribution of the data.

For the primary analysis, either a linear mixed model (LMM) analysis, or a series of repeated measures ANCOVA will be conducted to determine treatment effects in cognitive outcome as a function of allocated diet for each participant over time, and to assess whether high MedDiet intake over six months has a greater beneficial effect on age-related cognitive performance for older adults than HabDiet intake. For the secondary analysis, a series of bivariate Pearson correlations and a multiple mediation model using a bootstrapping approach will be conducted to assess whether the total and indirect effects of inflammatory, oxidative stress and metabolic biomarkers mediate the effect of diet (i.e. MedDiet and HabDiet) on age-related cognitive performance. The covariates included in the analysis will be organised into two models: Model 1. (gender, age, education, presence of Apolipoprotein E-ɛ4 allele); Model 2. (self-rated depressive symptoms, sleep quality, anxiety symptoms and stress levels).

#### Power and sample size calculation

Using the following standard conventions for estimating the sample size for a study with two equal groups (i.e. Z_α_ of 1.96 for 5% level of significance, Z_1-β_ [1-β% power] with β% of type II error [0.84 at 80% power], r = n1/n2 ratio for equal sample size for 2 groups and σ and d [the pooled standard deviation and difference of means of 2 groups]) it was estimated that a total sample size of 128 subjects (two groups of n = 64) would provide 80% power to detect a significant effect size of 0.5 (predicted change/SD of change, *p* < 0.05). These values were based from previous research [70]. Such statistical power referred to here represents the power required to detect a statistical difference between the treatment (MedDiet) and control (HabDiet) groups in terms of mean performance or variance in cognitive outcomes at an alpha level of .05, or put another way, the probability of rejecting the null hypothesis. This difference in change in cognitive outcomes would represent a meaningful outcome, and would be both clinically and theoretically relevant. To account for potential participant withdrawal, we aimed for an initial sample of 166 (an additional 38 volunteers, 19 per group) to allow for up to a 30% subject withdrawal rate.

## Discussion

The present protocol paper provides a detailed account of the design implemented for a 6-month, randomised, controlled, parallel group comparison intervention trial investigating the therapeutic potential for a Mediterranean dietary pattern to improve the cognitive performance and psychological well-being for healthy older adults aged 65 years and above. Advantages of the present study are: 1. the use of rigorous, randomised clinical intervention methodology, providing a stringent way of determining whether cause-effect relationships exists between the outcomes of interest, 2. investigating cognitively healthy older adults (≥65 years) as the population of interest, 3. the extensive set of outcomes investigated, including age-related cognitive, psychological well-being, cardiometabolic and genome health, and potential mediating factors, 4. the use of a comprehensive, psychometrically sound cognitive test battery, exclusively developed for MedDiet-cognitive ageing research, enabling assessment of a wide range of individual domains and aspects of cognition sensitive to normal ageing and diet. To our knowledge, this will be the first empirical study worldwide of this kind.

Undeniably, there is an urgent need for further empirical research to uncover novel strategies that may minimise such anticipated burden, promote healthy cognitive ageing, and help enhance the overall quality of life for older adults, dementia sufferers and their families and carers. Providing justified evidence regarding the efficacy of a MedDiet approach to safely reduce cognitive decline, and promote successful cognitive ageing could have great significance not only theoretically and scientifically on a global interface, but also for the overall health of society. Primarily, the outcomes of this research may have the greatest relevance for the estimated 524 million adults aged 65 years and older worldwide, who are most at risk of pathologic cognitive ageing [71], and also for the those among the Baby Boomers generation who are presently approaching the older age group (65 years and above). Furthermore, the implications of this research may also have great economic significance for society, Governments, and health care providers, if a potentially straightforward, safe, and inexpensive approach to maintain cognitive health can be met through modifying lifestyle and dietary behaviour.

## Abbreviations

AD: Alzheimer’s disease
ANCOVA: Analysis of covariance
APOE-4: Apolipoprotein E-ɛ4 allele
ARENA: Alliance for Research in Exercise, Nutrition and Activity
BMI: Body mass index
BVRT: Benton visual retention test
CES-D: The centre for epidemiological studies depression scale
CDT: Clock drawing test
CRTs: Plasma carotenoids
DEXA: Dual X-ray absorptiometry
DLQ: Diet and lifestyle questionnaire
DSB: Digit span backward
DSF: Digit span forward
ELF: Excluded letter fluency
EVOO: Extra virgin olive oil
FFQ: Food frequency questionnaire
FMD: Endothelial vasodilatory function
HabDiet: Habitual dietary pattern
HDL-C: Total cholesterol
*hs*CRP: C-reactive protein
ILF: Initial letter fluency
LDQ: Lifetime diet questionnaire
LMM: Linear mixed model
LNS: Letter-number sequencing
LSEQ: Leeds sleep evaluation questionnaire
MCA: Middle cerebral artery
MCAV: Middle cerebral artery blood flow velocity
MCI: Mild cognitive impairment
MedDiet: Mediterranean dietary pattern
MMSE: Mini-mental state examination
NATA: National association of testing authorities
PGF2α: F2-isoprostanes
PSS: Perceived stress scale
RAVLT: Rey auditory verbal learning test
RBCFA: Red blood cell fatty acid
RCTs: Randomised controlled intervention trials
SD: Standard deviation
SEM: Standard error of the mean
SOPs: standard operating procedures
STAI-Y: Spielberger State-Trait Anxiety Inventory Form Y
TCD: Cerebral vasodilator responses
TOL: Tower of London
UWA: University of Western Australia
WAIS-IV: Wechsler adult intelligence scale
